# Generating Pointing Motions for a Humanoid Robot by Combining Motor Primitives

**DOI:** 10.3389/fnbot.2019.00077

**Published:** 2019-09-18

**Authors:** J. Camilo Vasquez Tieck, Tristan Schnell, Jacques Kaiser, Felix Mauch, Arne Roennau, Rüdiger Dillmann

**Affiliations:** ^1^FZI Research Center for Information Technology, Karlsruhe, Germany; ^2^Karlsruhe Institute of Technology (KIT), Karlsruhe, Germany

**Keywords:** neurorobotics, motion generation, spiking neural networks (SNN), pointing a target, motor primitives, humanoid robot (HR), closed-loop

## Abstract

The human motor system is robust, adaptive and very flexible. The underlying principles of human motion provide inspiration for robotics. Pointing at different targets is a common robotics task, where insights about human motion can be applied. Traditionally in robotics, when a motion is generated it has to be validated so that the robot configurations involved are appropriate. The human brain, in contrast, uses the motor cortex to generate new motions reusing and combining existing knowledge before executing the motion. We propose a method to generate and control pointing motions for a robot using a biological inspired architecture implemented with spiking neural networks. We outline a simplified model of the human motor cortex that generates motions using motor primitives. The network learns a base motor primitive for pointing at a target in the center, and four correction primitives to point at targets up, down, left and right from the base primitive, respectively. The primitives are combined to reach different targets. We evaluate the performance of the network with a humanoid robot pointing at different targets marked on a plane. The network was able to combine one, two or three motor primitives at the same time to control the robot in real-time to reach a specific target. We work on extending this work from pointing to a given target to performing a grasping or tool manipulation task. This has many applications for engineering and industry involving real robots.

## 1. Introduction

The human motor system has been studied for a considerable period of time. Yet, robots lack robust, flexible and adaptive controllers comparable to the human motor system (Pfeifer and Bongard, 2006). One specific example is the capability to generate or pre-shape motions before execution (Shenoy et al., 2013).

Recent studies provide insights into the mechanisms for motion generation in the motor cortex. During reaching, activity in the motor cortex as a whole shows a brief but strong rotational component (Churchland et al., 2012; Russo et al., 2018). Instead of encoding parameters of movement in single neurons, the motor cortex as a whole can be understood as a dynamical system that drives motion. An initial state is produced externally and the system naturally relaxes while producing motor activity, which is then projected down the spinal cord to inter-neurons and motor-neurons (Churchland et al., 2012; Russo et al., 2018). Neural activity in the motor cortex shows a strong and amplified but stable response to initial activation (Hennequin et al., 2014). There is no broad consensus on the role of the motor cortex in voluntary movement. Nevertheless, neural correlates of many different types of parameters of arm movements have been found in the motor cortex (Kalaska, 2009). This behavior can be replicated by artificial neurons with strong recurrent connections balanced by strong inhibitory connections (Hennequin et al., 2014). Activity in the resulting network closely resembles activity in the motor cortex and can be used as an engine for complex transient motions (Hennequin et al., 2014). For example, in Ayaso (2001) an architecture detailing how to generate motor commands for arm motions is proposed, which also includes how learning and adaptation can be achieved by changing the gain.

A broadly accepted hypothesis is that the central nervous system uses linear combinations of a small number of muscle synergies to produce diverse motor outputs (Bizzi et al., 2008). The activation of the synergies can change based on sensor feedback to produce adaptive motions. The neuron activity in the intermediate zone of the spinal cord resembles motor primitives rather than individual muscles (Hart and Giszter, 2010). These neurons could act as building blocks for more complex voluntary movements. Different approaches have used the concepts of motor primitives to represent and model motions (Schaal, 2006; Tieck et al., 2018b,c). The *dynamic movement primitives* introduces a representation of movement as a spring-damping system in which the goal state is an attractor that allows for easily adaptable complex motor behaviors, both rhythmic and discrete (Schaal, 2006).

A set of approaches implemented with spiking neural networks (SNN) (Maass, 1997; Vreeken, 2003; Walter et al., 2016), represent motion using motor primitives to model target reaching (Tieck et al., 2018c, 2019) and different activation modalities (Tieck et al., 2018b). An SNN that autonomously learns to control a robotic arm through motor babbling and STDP was proposed in Bouganis and Shanahan (2010). In Chadderdon et al. (2012) an SNN is implemented that learns to rotate a single joint to a target and the learning is based on dopamine inspired reinforcement learning with a global reward and punishment signal. In Tieck et al. (2018a) a combination of reinforcement learning with a liquid state machine was used to learn continuous muscle activation of a musculo-skeletal arm.

To control robots in a way closer to biology we can use SNNs to implement models from neuroscience. Using the principles outlined in our previous work on motor primitives (Tieck et al., 2018b,c, 2019) and using the mechanisms for motion generation from the motor cortex (Ayaso, 2001; Hennequin et al., 2014), we can model pointing motions for a humanoid robot.

**Figure 1:**
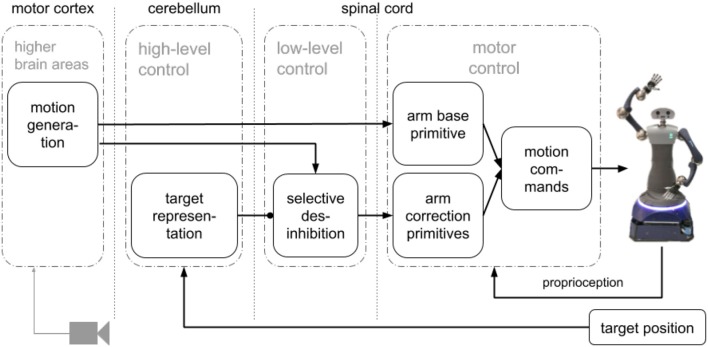
Architecture with main components: a motion generation layer that produces the activation patterns, a motor representation layer with motor primitives with a base and four corrections primitives, and a target representation layer to perform selective disinhibition.

We propose an SNN that combines a simplified model of the motor cortex to generate motions combining motor primitives to control pointing motions with a humanoid robot arm. Our approach for motion generation (pre-shaping) before execution has three main components (see Figure 1): a motion generation layer, a motor control layer with motor primitives and a target representation layer. The motion generation layer produces circular activity that creates the activation patterns for the primitives. The motor control layer has one base primitive for the pointing motion, and four correction primitives that point to targets left, right, up and down from the base motion target point. The target representation layer takes the target position and based on the relative distance to the base motion target point uses selective disinhibition to activate the correction primitives. We evaluated our approach with a humanoid robot, HoLLiE in Hermann et al. (2013), by defining different targets on a plane and having the robot point to them (see Supplementary Video 1).

## 2. Approach

Our SNN combines a simplified model of the motor cortex to generate motions combining motor primitives to control pointing motions with a humanoid robot arm. And here we present the details. In the work presented in Tieck et al. (2018c, 2019) we show how to perform online combination of primitives to achieve perception driven target reaching. In this work, the SNN performs motion generation (pre-planning) before execution using a bio inspired architecture.

We formalize the problem as follows: given an initial state of the robot and a set of primitives, move it to a target point on a plane. In classical robotics a system calculates the inverse kinematics (IK) and then validates the configuration to finally generate a motion trajectory. In contrast, our approach can do this without calculating the IK and without validating the resulting configurations. We define motor primitives for the arm as valid possible motions in the working space. The way new motions are generated is by using a base primitive that is activated, combined with a full or partial activation of the correction primitives. By using motor primitives to represent motions, we solve the trajectory generation in the “motor primitive space.” The resulting motions are combination of the primitives, which have no invalid configurations. In this work, we do not consider obstacles.

A go-cue in one neuron initiates circular activity in the motor generation layer that represents the motor cortex (Ayaso, 2001; Kalaska, 2009; Russo et al., 2018). The activity of this layer is used to activate the base and correction motor primitives (Tieck et al., 2018b,c, 2019). Based on an error signal representing the target, the correction primitives are disinhibited and combined with the base (Richter et al., 2012; Sridharan and Knudsen, 2015). The resulting spike activation is decoded to motor commands for the robot joints. The learned weights are the distance based inhibitory connections in motion generation layer, the connections to the base motor primitive, and the connections to the correction primitives. The architecture with the main components is presented in Figure 1. It has three main components: a motion generation layer, a motor control layer with motor primitives and a target representation layer.

The motion generation layer produces circular activity that creates the activation patterns for the primitives. A population generates neural activity over a certain period of time. The first step is to normalize spike activation by changing the weights of active neurons to get a similar amount of spikes from the whole population. Then, to obtain heterogeneity we add an inhibitory population with random connections.

The motor control layer provides the low level motor representation using motor primitives. There is one base motion primitive for pointing to the center, and four correction primitives that point to targets left, right, up and down from the base motion target point. The base primitive is activated and, depending on the target representation signal, the correction primitives are disinhibited.

The target representation layer takes the target position and, based on the relative distance to the base motion target point, uses selective disinhibition to activate the correction primitives. The target signal is the relative position to the base primitive final position, and it is used to regulate the activation of the correction primitives.

### 2.1. Motion Generation, M1

**Figure 2:**
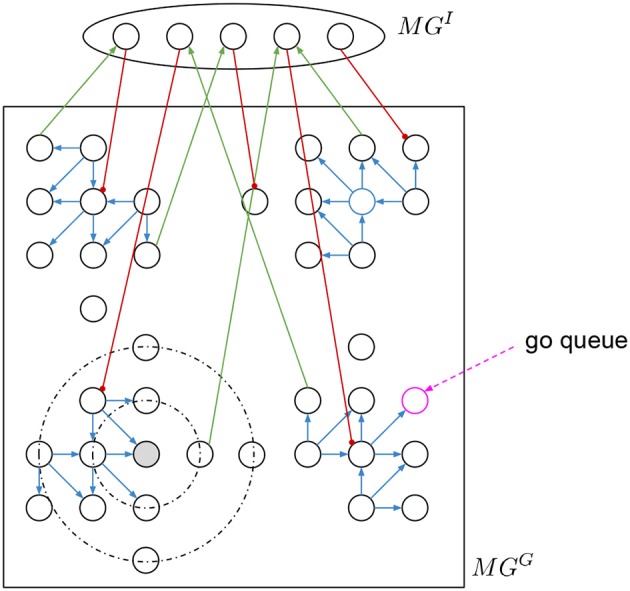
Motion generation layer with circular activation. A grid of neurons is connected with directed static excitatory connections depending on the quadrant. There are inhibitory plastic connections based on the distance. A hidden layer connected with input excitatory and output inhibitory connections to change the regular activity of the grid.

In the motion generation layer *MG* there is a group of two recurrent populations representing the motor cortex, one is a 2D grid *MG*^*G*^ and the other is an inhibitory *MG*^*I*^ to obtain heterogeneity (see Figure 2). This layer generates circular neural activity over a period of time (Churchland et al., 2012; Russo et al., 2018).

To initialize the motion generation layer there are two steps. First, we stabilize the spike activation in *MG*^*G*^ and second we add the inhibitory connections from and to *MG*^*I*^. Then we go over all neurons giving a go-cue to each one and we record how long the activity propagates. The go-cue is a continuous input of spikes to the respective neuron during 10 ms. For each motion we select the “go-neuron” as the neuron that produces activity with similar time to the desired motion.

*MG*^*G*^ is square grid of 20 × 20 neurons with recurrent connections (see Figure 2). There are two types of connections, the directed excitatory to create the circular activity and the local inhibitory to stabilize the activity. The excitatory connections (blue connections in Figure 2) are static and have specific directed connectivity depending on the quadrant the neurons area to amplify the activity and force the rotational activation. The distance based local inhibitory connections (black dotted circular lines in Figure 2) stabilize the activity.

To normalize the spike activity of *MG*^*G*^, the inhibitory weights are changed to achieve a specific total activity $MG_{norm}^{G}$ with the following learning procedure. We add a spike recorder to all *MG*^*G*^ neurons. A go-cue (pink dotted arrow in Figure 2) is given as short burst of 10 ms of spikes into one single neuron at a time. This initial neuron is chosen randomly every time, so that there are no “dark” spots in *MG*^*G*^ without spike activity. Every 100 ms Δ*t* (nest.sim(100 ms)) the simulation is stopped. The total spikes of *MG*^*G*^ in that δ*t* are counted as $MG_{spikes}^{G}$. If $MG_{spikes}^{G}>MG_{norm}^{G}$, then increase the weights by Δ*w* of the inhibitory connections coming out of all active neurons. Else if $MG_{spikes}^{G}<MG_{norm}^{G}$, then decrease them. The Δ*w* must be small, so that a weight update does not kill the activity. In other words, we want to regulate the global total activity of the *MG*^*G*^ population, if it is too high then propagate less, if it is too low then propagate more.

After training, once the circular activity propagation of *MG*^*G*^ is stable, we add a small population *MG*^*I*^ with random input and output connections to and from the 2D grid *MG*^*G*^ to obtain heterogeneity. Both, input and output connections are static and random. The output connections—from *MG*^*I*^ to *MG*^*G*^—are strong inhibitory (red connections in Figure 2), and the input connections are excitatory (green connections in Figure 2). To set the connections, we set fix numbers of input and output connections, then we sample random neurons from both populations and connect them.

With *MG*^*I*^ and *MG*^*G*^ connected, we then go over all neurons in *MG*^*G*^ to asset the resulting activity. We give again a “go-cue” as short burst of spikes for 10 ms into each single neuron “go-neuron” (pink circle in Figure 2), and then measure how long does the activity propagates. The time is measured either until no more spikes occur, or interrupted after a maximum time limit in simulation steps. The activity duration for each “go-neuron” is stored in a table. Then we pick those with similar time to the desired motions, and this will be the “go-neuron” for the primitives.

### 2.2. Base and Correction Motor Primitives

**Figure 3:**
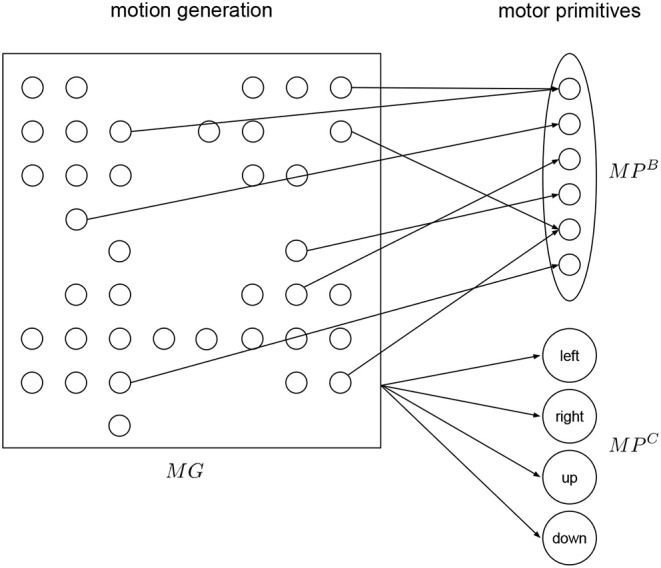
Motor primitives layer. The base primitive is detailed for three joints, the same structure applies to the correction primitives. There are four correction primitives—left, right, up, and down. All primitives receive activation input from the motion generation layer.

The motor primitive layer *MP* is a layer for low level motor representation using motor primitives (Tieck et al., 2018b,c, 2019) (see Figure 3). The primitives are combine to generate a specific motion activated by the motion generation layer *MG*. In *MP* there are populations, one for the base primitive and one for each of the correction primitives. During execution of a motion, the base primitive is activated, and depending on the target representation signal, the correction primitives are activated.

To generate pointing motions in a certain working space, we define the following motion representation. We define first a base primitive *MP*^*B*^ (see Figure 3), which is a motion to point at the center of the working space. Then we define four correction primitives *MP*^*C*^ to point at points to the left, right, up and down of the center (see Figure 3). This four points define an ellipsoid as the boundary of the working space in the plane.

For each primitive, a different population is connected to *MG*. Each primitive has two motor neurons per joint in the robot. Each output spike causes small change in the corresponding robot joint, it is defined as a fixed gain factor that regulates the speed. There is a detailed view of the primitive population for the base motion in Figure 3. The training is done one by one to resemble the exemplary motion. We use supervised learning to minimize the error and adapt the weights and produce a specific motion (Tieck et al., 2018b).

### 2.3. Target Representation

**Figure 4:**
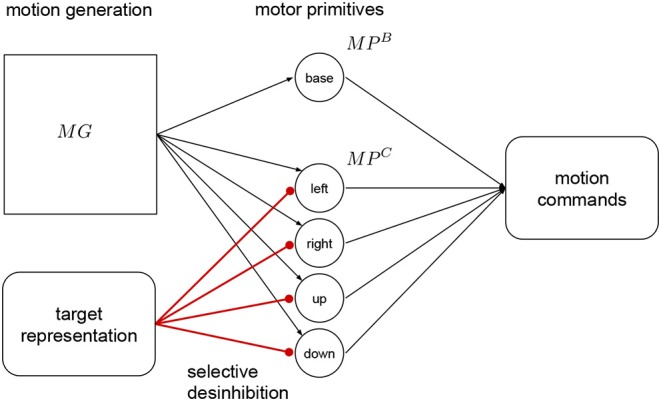
Target representation. This layer projects with strong inhibitory connections to the correction primitives The target is represented as a relative error signal to the target of the base primitive. This signal is used to disinhibit the primitives, and adapt the resulting motion. The output from the base primitive and the active correction primitives is combined to produce the motion commands for the robot.

The target representation layer is connected to the correction primitives with inhibitory synapses as shown in Figure 4. The correction primitives are inhibited by default, and they are disinhibited according to required adaptation provided by this layer. This mechanism is called selective disinhibition and it is used for attention mechanisms, decisions and mechanisms for target selection (Richter et al., 2012; Sridharan and Knudsen, 2015). For example, if no correction to the right is necessary, then the right primitive remains fully inhibited. In Kawato (1999) and Wolpert et al. (1998), they see the cerebellum as an internal model that can predict how the end result of a known motion will be like. This prediction can be compared to a desired target to make the respective corrections before execution.

In our approach we use a relative target representation, with the target's relative position to the base primitive final position. That signal is used to regulate the activation of the of the neurons in this layer, by decreasing the input current proportionally this layer activates the correction primitives using selective disinhibition. This signal translates to the amount or percentage of activation, between 0 and 1, of the respective correction primitives, with 1 being full inhibition and 0 full activation. This adaptation or pre-shaping happens before executing the motion.

## 3. Results

**Figure 5:**
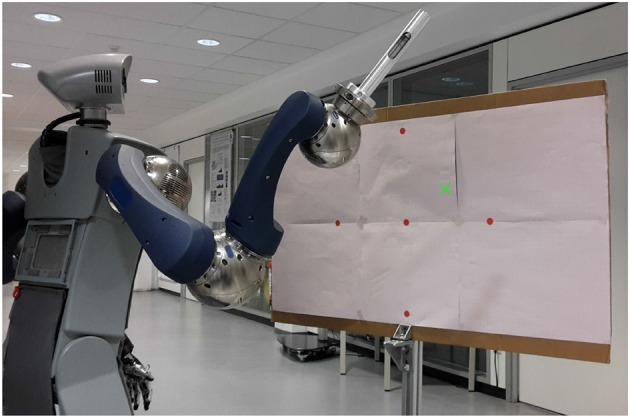
Basic experiment setup. The robot is in a starting position in front of the board plane and will produce a motion toward a target point (*green x*). Red points show the targets for the base and correction primitives that are already learned.

In most modern and more complex robotic applications motions have to be dynamically generated according to flexible targets or constraints. A major component of many robot tasks is the reaching of specific, often dynamic targets. While this is usually followed by some form of manipulation of an object, the pure act of reaching a specified goal state with a robot manipulator can be understood as a pointing motion. Due to this, we use pointing toward different goal-points on a board plane (see Figure 5) to evaluate how well the robot generates adaptive motions.

### 3.1. Experiment Setup

Initially, base motor primitives have to be learned. A base motion of pointing toward a central target is manually created, but could easily be generated with motion capture or teached-in. The network is then trained to produce this specific pointing motion when all correction primitives are fully inhibited. Afterwards, the base motion is manually adapted toward 4 specified points in the target area, each with a distance of 25 cm from the center to the left, right, top and bottom (red points in Figure 5). The correction primitives in the network are trained to produce the difference from the base motion toward these adapted motions, so that as a whole the network produces them when their the corresponding correction primitive is uninhibited.

This allows the network to create different motions by partially inhibiting the corrections primitives. The quality of the generated motions is measured based on the network's ability to point at different targets. The reference points are used as a coordinate system, with positive x-axis representing the inverse inhibition of the right primitive and the negative axis the left primitive, respectively. In the same way, the y-axis represents the up and down primitives. This allows a mapping from every point on the board to specific inhibitions of the correction neurons. A motion is generated with these inhibitions set manually and the final position of the end-effector of the robot is then compared with the intended goal. The distance between actual and target position is used as a measure of error in the following experiments.

### 3.2. Humanoid Robot HoLLiE

HoLLiE, Hermann et al. (2013) is a mobile service robot with two functional arms and humanoid hands (see Figure 5). The robot was developed at the FZI Research Center for Information Technology for different tasks, such as accompanying visitors and mobile manipulation (see^1^). With a range of different sensors and a highly articulated body HoLLiE can handle everyday objects, interact with humans in multiple ways and therefore be employed in various service robotic scenarios. For these characteristics HoLLiE was chosen to achieve human-like pointing motions, as the arms are mounted on an upper body in a similar kinematics to a human arm.

### 3.3. Implementation Details

Motions are generated by an SNN using the PyNN API implemented in NEST, Diesmann and Gewaltig (2001) running on a laptop computer. We use *Robot Operating System (ROS)*^2^ as a communication layer to connect NEST with the robot.

The SNN was simulated in steps of 100 ms and the spikes in this time frame were accumulated before being sent to the robot. This frequency is enough to generate smooth real-time robot movements, and a complete pointing motion takes about 10s. The generated spikes in the output of the motor-neurons were directly decoded into changes in joint values for the robot. The neuron activity is decoded by changing joint position by a fixed value for each spike. The resulting joint values were than used as goals for the joint trajectory controller in ROS.

During training of *MG*^*G*^, the weights of one iteration are stored in a dictionary data-structure where all the required weight updates are performed. Only after all updates have been calculated, the “set weights” function in NEST is called, as constant weight changes are greatly reduce the simulation time for little gain. Using this, the total training time could be reduced to about 1 h on a single processor.

The network is implemented with basic leaky integrate and fire neurons LIF. The layer *MG* is built as a population organized in a grid of 20 × 20 neurons *MG*^*G*^ and an inhibitory population of 20 neurons *MG*^*I*^. For each of the 5 motor primitives *MP* (one base and four correction) 2 neurons are used per joint, with three active joints being used for the evaluated motions, for a total of 30 neurons. The total SNN contains 450 neurons and about 20,000 synapses.

### 3.4. Experiment

**Figure 6:**
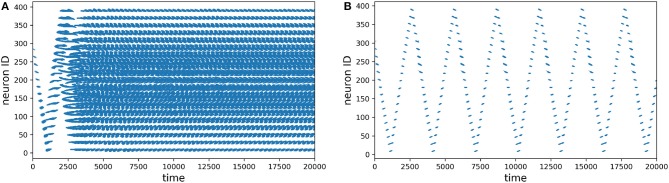
Spike plots for the motion generation population, time is in milliseconds. **(A)** Before learning. **(B)** After learning.

The first thing we evaluated was how does the learning in the network work, specially in the motion generation layer. In Figure 6 we recorded the spike activity of all the neurons before and after learning. Without learning, you can see on the left how the go-cue propagates in the neurons and then saturates, producing chaotic activation. After learning, you can see on the right how the activation of the population is periodic (circular) and is stable.

**Figure 7:**
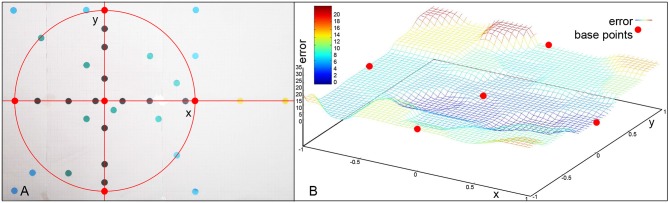
**(A)** Different points evaluated in the experiments. **(B)** Error values over target area and error values for learned base points (red). Outside of the circular area encapsulated by the base points, the error increases significantly.

**Figure 8:**
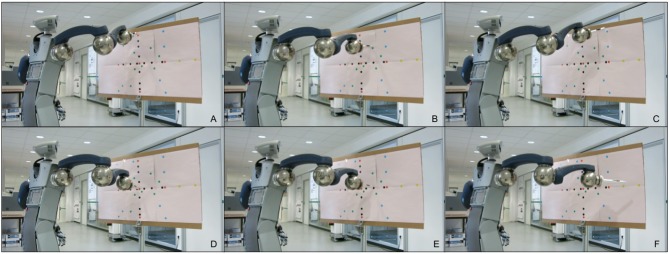
Frame sample of the experiments. This shows the robot pointing at different types of targets on the board in Figure 7A.

Throughout the experiments different types of targets were attempted to be reached based on the board displayed in Figure 7. The distance from the target in millimeters is used as an error for evaluation. The base motion is the center red dot. The correction primitives are the red dots on the circle. If we only use one of the correction primitives at a time, we obtain black dots. A combination of multiple correction primitives are the green dots. The blue dots are outside of the working space, but still in the primitive space. The yellow dots on the right are extrapolations. The frame sample in Figure 8 shows the robot pointing at different types of targets on the board.

**Figure 9:**
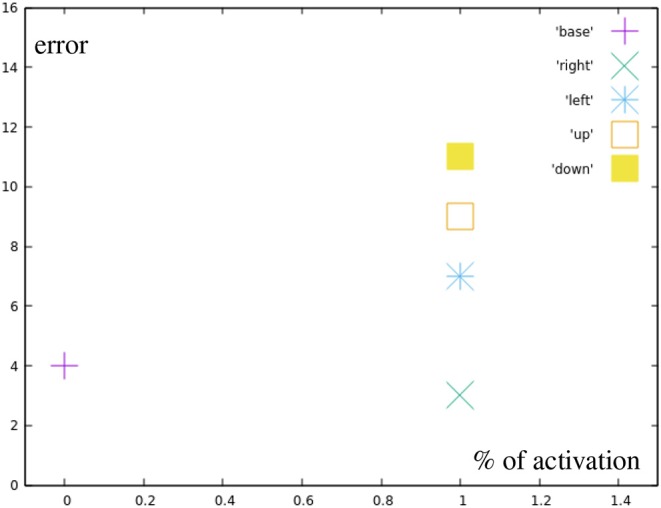
Error by distance to center for reaching all the base points (red in Figure 7A).

Red points represent the targets for the manually designed base motions that can be reached by fully inhibiting all or all but one correction primitives. Figure 9 shows the errors for the different base motions. It can be seen, that they are not hit completely accurately, which results from the relatively high impact single spike inaccuracies have on the end position.

**Figure 10:**
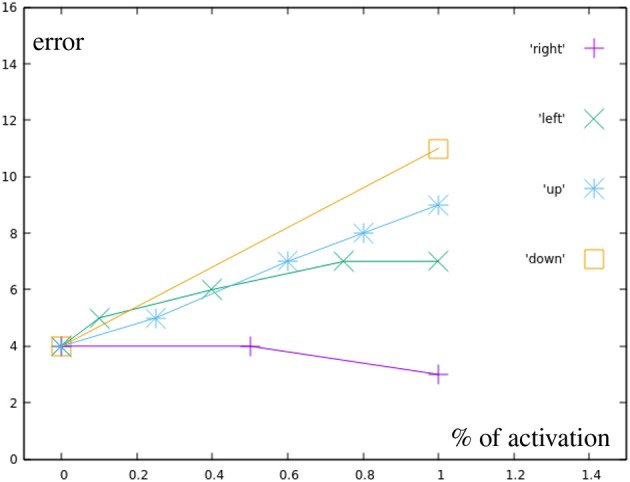
Error by distance to center for different correction primitives (black in Figure 7A). Right is the most accurately learned primitive, down the least accurate one.

The black points represent motions using only a single, partially inhibited correction primitive. Figure 10 shows that there is no additional error created by partially inhibiting the primitives, other than the already existing inaccuracy in the learned motions themselves. Green points display motions combining two correction primitives, but with a total distance from the base motion not greater than one full correction primitive.

**Figure 11:**
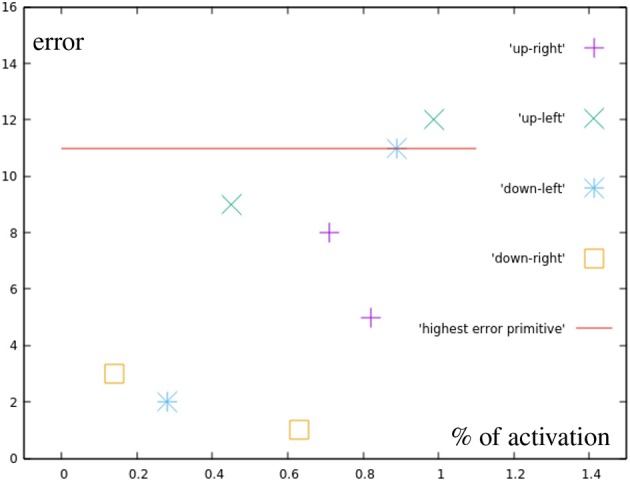
Error by distance to center for points in the different quarters (green in Figure 7A). For comparison, the error of the most inaccurate correction primitive is noted.

While Figure 11 can not show as easily how the in these targets results purely from the base primitives, with the exception of one point directly on the circular test area all motions produced a smaller error than the most incorrect base motion. This again suggests, that no additional error is added through the combination of two correction primitives. The light blue points are also created by combining two correction primitives, in this case, though, their distance to the base is greater than one the distance of one primitive.

**Figure 12:**
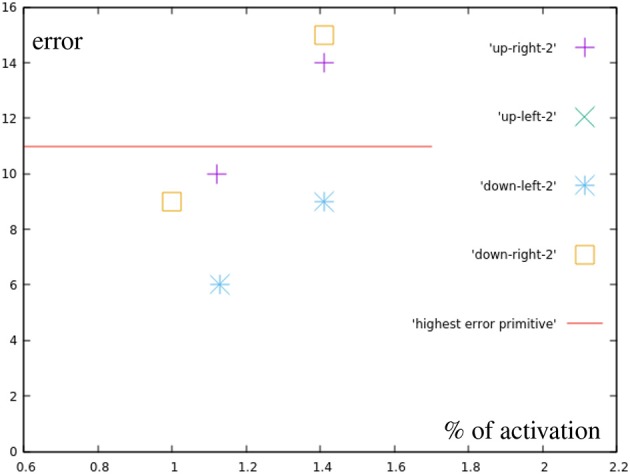
Error by distance to center for points in the different quarters (blue in Figure 7A). For comparison, the error of the most inaccurate correction primitive is noted.

These results (Figure 12) show errors that do not seem to simply happen from inaccuracies in the learned motions. The upper right point using both primitives fully, for example, generates an error of 14 millimeters, while the sum of the errors of both used correction primitives is only 12 mm.

**Figure 13:**
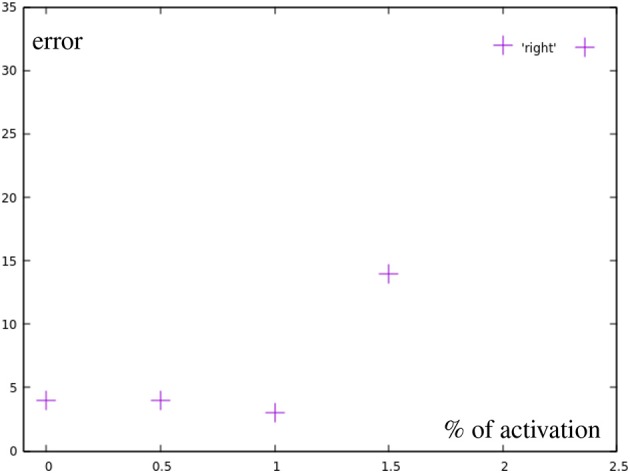
Error by distance to center for motions using more than one full primitive (yellow in Figure 7A). Points after 1.0 show a greatly increased error.

All marked points are well within the workspace of the robot. But yellow points are not reachable with the defined primitives, meaning an activation of 1 (100) of any combination of the motions will not go outside of the bounds defined by the primitives (red dots). Moreover, outside of the circular area used in the previous experiment the method of combining primitives loses precision, as a consequence. Finally, the yellow points are actually unreachable by combining primitives with total activation. An extrapolation from the right primitive would be necessary. To accomplish this, the right primitive is not only uninhibited, but additional spikes are added to generate more activity. So, there is a correlation of the error with the positions of the learned base motions. As Figure 13 shows, while direction of the adaptation is correlated, the error is greatly increased and a precise correction does not occur. The total errors over the target area can be seen in Figure 7B. In a circular area between the base motions, the error can be reduced to inaccuracies in learning, while outside of this area additional errors can be measured.

## 4. Discussion

Based on the results and the evaluation form the experiments we can highlight certain aspects. If the target distance is of one correction primitive or less, then there is no significant added error through adaptation. If there is a higher distance, then the error increases. The error gets a relatively high impact from single spikes and a reduction by using larger populations and different encoding techniques would allow for more precision. This is a low level control problem, and we currently work on a spike based controller for ROS to achieve smooth control.

We successfully implemented and tested an SNN for voluntary adaptive motions using an architecture based on recent theories about motion generation in the central nervous system. The network was able to pre-shape motions and generate new trajectories before the execution by combining primitives using selective disinhibition. The SNN was able to control a real humanoid robot in real-time in a closed-loop scenario. This approach can be used with different robot arms, and is not dependent on a specific kinematic structure.

In the future we want to benchmark the technical aspects, and increase the precision and speed of the motions. With the recent advances in backpropagation-like learning rules for SNN as in Kaiser et al. (2019), we can learn different motion types for different tasks in same network, and start them with different go-cues. We also want to integrate event-based vision to this system to get the target and drive the adaptation as in Kaiser et al. (2016), and to explore learning by demonstration as in Kaiser et al. (2018). We work on extending this work form pointing to a given target to perform there a grasping or tool manipulation task. This has many applications for engineering and industry with real robots.

## Data Availability

All datasets analyzed for this study are included in the manuscript and the Supplementary Files.

## Author Contributions

All authors participated in writing the paper. JCVT, TS, JK, and FM conceived the experiments and analyzed the data.

### Conflict of Interest Statement

The authors declare that the research was conducted in the absence of any commercial or financial relationships that could be construed as a potential conflict of interest.

## Footnotes

^1^https://www.fzi.de/en/research/projekt-details/hollie/

^2^http://www.ros.org/
